# Editorial Comment: TClassification of the renal papillary abnormalities by flexible ureteroscopy: evaluation of the 2016 version and update

**DOI:** 10.1590/S1677-5538.IBJU.2022.02.05

**Published:** 2022-03-01

**Authors:** Alexandre Danilovic

**Affiliations:** 1 Universidade de São Paulo Hospital das Clínicas, Faculdade de Medicina Departamento de Urologia São Paulo SP Brasil Departamento de Urologia, Hospital das Clínicas, Faculdade de Medicina da Universidade de São Paulo, São Paulo, SP, Brasil

Christophe Almeras 1, Michel Daudon 2, Vincent Estrade 3, Jean Romain Gautier 4, Olivier Traxer 5, Paul Meria 6

World J Urol. 2021 Jan;39(1):177-185.

## COMMENT

Endoscopic view of the collecting system provides the chance to observe many abnormalities that could be correlated to urinary stone formation. Papillary calculi result from subepithelial lesions (1). Therefore, the observation of papillary abnormalities could help to understand lithogenesis and eventually help the management of patient's treatment.

Almeras et al. reported the endoscopic evaluation of renal papillae during 88 consecutive flexible ureteroscopies based on the 2016 proposed classification (2). This classification was inspired on the oncologic TNM classification. It included stone description (Sx), number and type of papillary abnormality (nPx) and the amount of Randall's plaque (Rx) (3). The present study updated the former classification to new SxnPxDrx/i/px, including mixed type of stone, excluding subepithelial stones, including papillary abnormalities of medullary sponge kidney and including description of deposits (D) of the amount of Randall's plaque (r), intrapapillary deposits (i) and intraductal plugs (p). Main findings were that 83% of the patients had Randall plaques and only 4.5% of the patients had no abnormalities. Erosions were present in 55.7%, anchored stones in 30.7%, intraductal crystallization in 15.9% and extrophic papillae in 8%. The description of the renal papillae showed clinical importance because it was correlated to the diagnosis of a metabolic lithogenesis. High amount of Randall's plaque was associated with dark anchored stones (Sa1). Calcium phosphate stones were correlated to intraductal crystallization (Sc) and hypercalciuria was higher in light anchored stones (Sa2) than dark anchored stones (Sa1).

As endoscopic surgeons, we should seize the opportunity to use the endoscopic view not only to treat the already formed stones but also to help patients with the diagnosis of the cause of stone formation. The proposed classification by Almeras et al. seems to be too complex to be adopted by urological community and should be validated by other investigators to be recommended but it is the most embracing endoscopic classification so far.
